# Proteomic Analysis of Protective Effects of *Epimedium* Flavonoids against Ethanol-Induced Toxicity in Retinoic Acid-Treated SH-SY5Y Cells

**DOI:** 10.3390/molecules27031026

**Published:** 2022-02-02

**Authors:** Xiaohua Yang, Huafeng Zhang, Lu Li, Xuexue Zhou, Yichao Liu, Jianghua Lai

**Affiliations:** 1 Health Science Center, Xi’an Jiaotong University, Xi’an 710061, China; xhyang@mail.xjtu.edu.cn; 2 International Joint Research Center of Shaanxi Province for Food and Health Sciences, National Engineering Laboratory for Resources Development of Endangered Crude Drugs in Northwest China, Provincial Research Station of Se-Enriched Foods in Hanyin County of Shaanxi Province, College of Food Engineering and Nutritional Science, Shaanxi Normal University, Xi’an 710062, China; liluacademic@yahoo.com (L.L.); xuexzhou@snnu.edu.cn (X.Z.); ryuyichao@snnu.edu.cn (Y.L.)

**Keywords:** alcohol, proteomics, *Epimedium* flavonoids, retinoic acid-treated SH-SY5Y cells, alcoholism, mechanism of action

## Abstract

Alcohol (ethanol) is one of the most common addictive psychoactive substances in the world, and alcoholism may result in harmful effects on human health, especially on the nervous system. Flavonoids are regarded as the main active constituent in *Epimedium*, which has been used to cure some nervous system diseases such as amnesia for over 1000 years. Here, the protective effects of *Epimedium* flavonoids against ethanol-induced toxicity in retinoic acid (RA)-treated SH-SY5Y cells were investigated. Their mechanism was explored by a label-free proteomic approach combined with bioinformatic analysis for the first time. The results showed that ethanol treatment decreased cell viability by 18%, whereas the viability increased significantly after intervention with *Epimedium* flavonoids (*p* < 0.01). According to proteomic and bioinformatic analyses, hundreds of differentially expressed proteins (DEPs) were identified and classified as biological process (GO_BP), cellular component (GO_CC) and molecular function (GO_MF). Among them, GO_MF of DEPs, especially molecular function relevant to G proteins, greatly changed in SH-SY5Y cells pretreated by *Epimedium* flavonoids. In the alcoholism pathway, the expression of the Gi protein was up-regulated under the influence of ethanol, whereas *Epimedium* flavonoids could reverse the expression profile, both of which were validated by Western blot assay. In conclusion, Gi protein seemed to be an important factor in the alcoholism pathway to suppress the ethanol-induced toxicity of SH-SY5Y cells. These findings suggest a protective potential of *Epimedium* flavonoids against ethanol-induced toxicity to neurons via the regulation of Gi protein function.

## 1. Introduction

Alcohol (ethanol) is one of the most common addictive psychoactive substances in the world [1,2]. Excessive consumption of alcohol might result in detrimental effects on human health, such as alcoholic liver diseases, cancers and neuropsychiatric disorders [1,3,4]. Additionally, toxic effects on the nervous system are regarded as one of the most significant health impacts caused by alcohol intake [3]. In particular, excessive alcohol intake considerably affects neurotransmitter (e.g., dopamine and acetylcholine) metabolism, reduces the number of neurons, induces neurotoxicity, inhibits neurogenesis, causes neuron apoptosis, suppresses nerve signals, or even leads to impairment of the central nervous system [5,6,7,8,9]. Therefore, it is of paramount importance to develop effective medicines to alleviate ethanol-induced toxicity to the nervous system.

It is reported that a vast array of Chinese medicinal materials (e.g., *Pueraria lobata*, *Lablab purpureus*) were applied for the prevention and treatment of alcohol dependence or alcoholism [10,11]. Pharmacological studies indicated that several flavonoids, obtained from traditional medicinal materials and their related derivatives, exhibited therapeutic potential against alcohol dependence and alcoholism [4,11,12]. *Epimedium*, a traditional Chinese medicine rich in flavonoids, was used to improve memory and cure amnesia, as recorded in famous ancient Chinese medicinal documents such as *Shennong’s Classic of Materia Medica*, *Ri Hua Zi Ben Cao* and *Compendium of Materia Medica*. Nowadays, *Epimedium* is used to treat neuronal injury, promote neuronal growth, improve impaired neuronal function and so on [13,14,15,16,17,18]. However, there are no reports on the application of *Epimedium* flavonoids for the prevention and treatment of alcohol dependence and alcoholism, as well as the corresponding mechanisms.

Proteomic analysis is one of the modern biomedical techniques that is increasingly used to unveil the mechanisms of action and drug targets of bioactive constituents (e.g., ganoderic acid, tanshinone, salvianolic acid and berberine) in traditional Chinese medicine [19,20,21,22,23]. Compared to conventional iTRAQ (isobaric tags for relative and absolute quantification)-based proteomics, label-free proteomics have distinctive advantages, including a simplicity of sample preparation, low cost of reagents and high fidelity of protein samples [24]. In this study, the protective effects of *Epimedium* flavonoids against ethanol-induced toxicity in retinoic acid (RA)-treated SH-SY5Y cells were investigated. Their mechanism was also explored by a label-free proteomic approach combined with bioinformatic analysis. Furthermore, the impact of selected proteins on the alcoholism pathway was validated by a Western blot assay.

## 2. Results

### 2.1. Protective Effects of Epimedium Flavonoids on RA-Treated SH-SY5Y Cells Exposed to Ethanol

**Figure 1:**
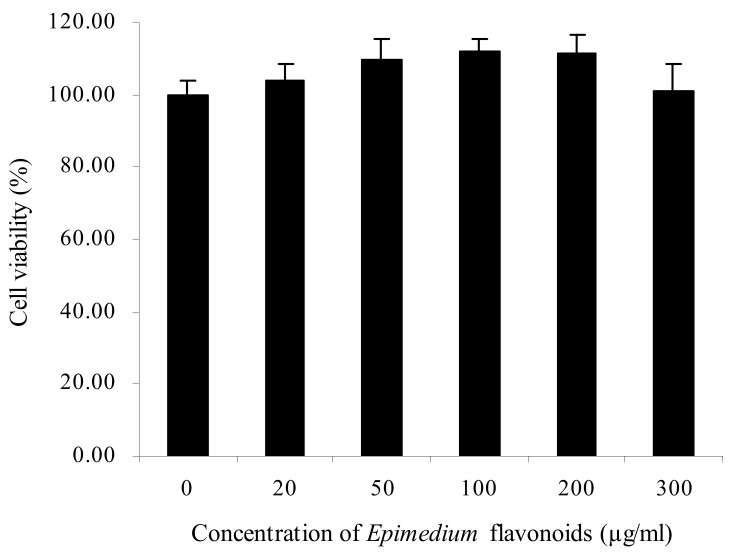
Cytotoxicity of *Epimedium* flavonoids on RA-treated SH-SY5Y cells. Data were expressed as means of five replicates (*n* = 5).

**Figure 2:**
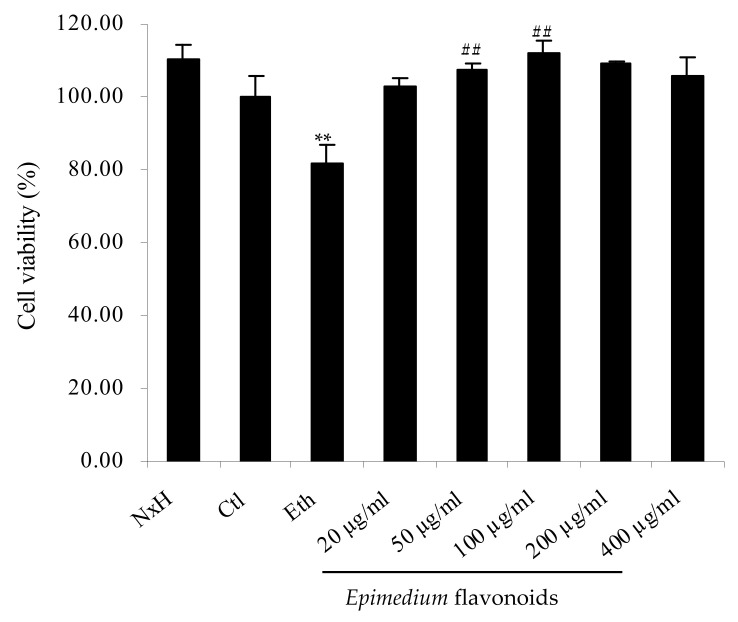
Protective effects of *Epimedium* flavonoids on RA-treated SH-SY5Y cells under ethanol exposure. Ctl: control group; Eth: ethanol group; NxH: naloxone hydrochloride group; 20 μg/mL, 50 μg/mL, 100 μg/mL, 200 μg/mL and 400 μg/mL represent flavonoids group containing the corresponding concentrations of *Epimedium* flavonoids. Data were expressed as means of five replicates (*n* = 5). **: *p* < 0.01 (versus control group); ^##^: *p* < 0.01 (versus ethanol group).

As shown in Figure 1, the viability of RA-treated SH-SY5Y cells rose with the increase in *Epimedium* flavonoids concentration from 20 to 200 µg/mL, and decreased thereafter (from 200 to 300 µg/mL). There was no significant difference of cell viability between the control group and flavonoids group at various concentrations (20–300 µg/mL) (*p* > 0.05). *Epimedium* flavonoids showed no cytotoxicity and could promote the proliferation of RA-treated SH-SY5Y cells to some extent. Taking into account the physiological influence of ethanol on humans, 200 mM was chosen as the inducing dose of ethanol [5]. The protective effects of *Epimedium* flavonoids on RA-treated SH-SY5Y cells damaged by ethanol are presented in Figure 2. Clearly, ethanol treatment decreased cell viability by 18% (*p* < 0.01). When the cells were pretreated by *Epimedium* flavonoids prior to ethanol addition, cell viability increased significantly in comparison to the ethanol group (*p* < 0.01), and was equivalent to the control group (*p* > 0.05). In the flavonoids group, with the increase in flavonoids concentrations, cell viability first increased and then decreased slightly. Cell viability in flavonoids group at a concentration of 50 µg/mL was 30.86% higher than that in the ethanol group (*p* < 0.01). Accordingly, cells treated with 50 µg/mL flavonoids were used for subsequent proteomic experiments and a Western blot analysis. It can be concluded that *Epimedium* flavonoids notably protected RA-treated SH-SY5Y cells against alcohol damage.

### 2.2. Influence of Epimedium Flavonoids on Protein Profile in RA-Treated SH-SY5Y Cells Exposed to Ethanol

Label-free proteomics were employed to explore the mechanism underlying the protective effects of *Epimedium* flavonoids against ethanol-induced toxicity in RA-treated SH-SY5Y cells. Differentially expressed proteins (DEPs) between the control group, ethanol group and flavonoids group were analyzed. To ensure authentic identification and quantification, a minimum of seven amino acids in each peptide and one unique peptide in each protein were required. Additionally, the maximum false discovery rate (FDR) was set at 1% [25]. There were 2829, 2195 and 2537 peptides in the control group, ethanol group and flavonoids group, respectively; more than 1000 proteins were identified (FDR < 1%) in each group. Among them, 126 differentially expressed proteins were detected between the control group and ethanol group. The expression of 68 DEPs was down-regulated, while the expression of 58 DEPs was up-regulated. Between the ethanol group and flavonoids group, 122 differentially expressed proteins were found. Among them, the expression of 62 DEPs was down-regulated, whereas the expression of 60 DEPs was up-regulated.

### 2.3. Functional Annotation and Classification of Differentially Expressed Proteins

**Figure 3:**
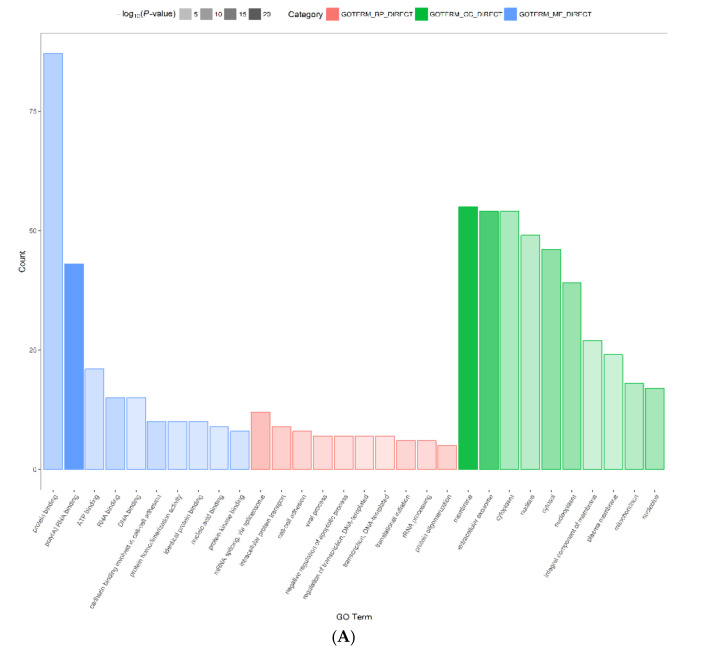

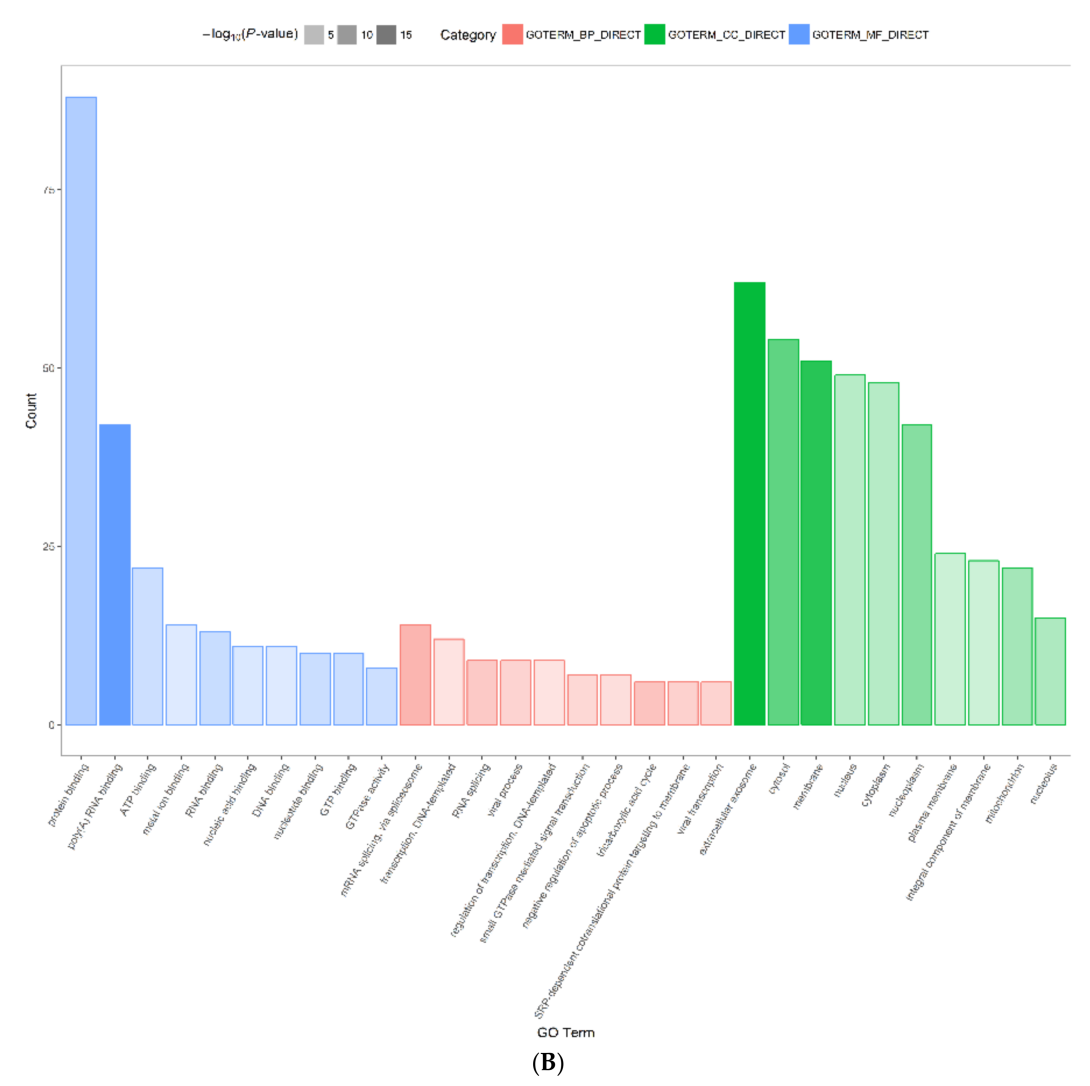
Gene ontology analysis of differentially expressed proteins. (**A**) Ethanol group versus control group; (**B**) flavonoids group versus ethanol group. GOTERM (GO)_MF (blue column) represents molecular function; GOTERM (GO)_CC (green column) represents cellular component; GOTERM (GO)_BP (pink column) represents biological process.

In order to understand the functions of all differentially expressed proteins, they were analyzed based on gene ontology (GO). Additionally, DEPs were classified as biological process (GO_BP), cellular component (GO_CC) and molecular function (GO_MF) (Figure 3). According to the molecular function in Figure 3, the majority of DEPs between the control group and ethanol group were involved in protein-binding (87 proteins, 69.6% ratio), poly(A) RNA binding (43 proteins, 34.4% ratio), ATP binding (21 proteins, 16.8% ratio), RNA binding (15 proteins, 12.0% ratio), DNA binding (15 proteins, 12.0% ratio), cadherin binding relevant to cell–cell adhesion (10 proteins, 8.0% ratio), and protein homodimerization activity (10 proteins, 8.0% ratio). Among them, protein-binding (87 proteins, 69.6% ratio) was intimately linked to G proteins. Moreover, G proteins were associated with other GO_MF such as GTP binding (8 proteins, 6.4% ratio), metal ion binding (8 proteins, 6.4% ratio), GTPase activity (7 proteins, 5.6% ratio), signal transducer activity (4 proteins, 3.2% ratio), GDP binding (3 proteins, 2.4% ratio), protein domain-specific binding (3 proteins, 2.4% ratio) and G protein-coupled receptor binding (2 proteins, 1.6% ratio).

The majority of DEPs between the ethanol group and flavonoids group were involved in protein binding (88 proteins, 72.7% ratio), poly(A) RNA binding (42 proteins, 34.7% ratio), ATP binding (22 proteins, 18.2% ratio), metal ion binding (14 proteins, 11.6% ratio), RNA binding (13 proteins, 10.7% ratio), nucleic acid binding (11 proteins, 9.1% ratio), DNA binding (11 proteins, 9.1% ratio), nucleotide binding (10 proteins, 8.3% ratio), and GTP binding (10 proteins, 8.3% ratio). Among them, protein binding (88 proteins, 72.7% ratio) was highly correlated with G proteins. Additionally, G proteins were related to other GO_MF such as GTPase activity (8 proteins, 6.6% ratio), protein complex binding (6 proteins, 5.0% ratio), GTPase binding (2 proteins, 1.7% ratio), signal transducer activity (2 proteins, 1.7% ratio), alkylglycerophosphoethanolamine phosphodiesterase activity (1 protein, 0.8% ratio), spectrin binding (1 protein, 0.8% ratio) and type 1 angiotensin receptor binding (1 protein, 0.8% ratio). These results indicated that the GO_MF of DEPs, especially molecular function related to G proteins, greatly changed in SH-SY5Y cells pretreated by *Epimedium* flavonoids.

### 2.4. Analysis of DEPs Related to Alcoholism

**Figure 4:**
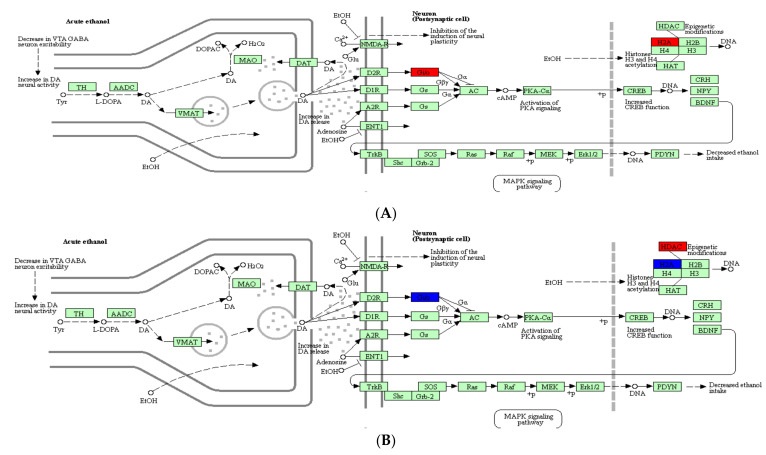
KEGG analysis of alcoholism pathway in ethanol and flavonoids groups. (**A**) Ethanol group versus control group; (**B**) flavonoids group versus ethanol group.

In order to further elucidate the impact of *Epimedium* flavonoids on the main pathways and the key factors in ethanol-induced SH-SY5Y cells, DEPs were categorized using the Kyoto Encyclopedia of Gene and Genomes (KEGG) website (Figure 4). The results indicated that DEPs participated in many pathways, such as the alcoholism pathway, metabolism-related pathway and dopaminergic synapse pathway. According to the alcoholism pathway depicted in Figure 4, ethanol exposure was closely associated with the Gi protein, histone H2A and histone deacetylase (HDAC). Among them, the Gi protein was linked to ethanol intake via a series of downstream proteins. Under the influence of alcohol, the expression of Gi protein was up-regulated. On the contrary, the expression of Gi protein was down-regulated after the intervention with *Epimedium* flavonoids (Figure 4).

### 2.5. Validation of Selected Proteins by Western Blot

**Figure 5:**
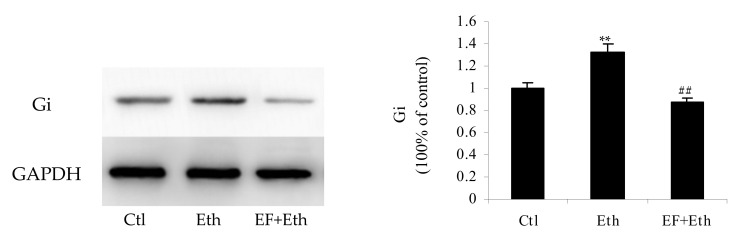
Western blot analysis of Gi protein. Ctl: control group; Eth: ethanol group; EF + Eth: flavonoids group. **: *p* < 0.01 (versus control group); ^##^: *p* < 0.01 (versus ethanol group).

According to the above-mentioned proteomic analysis, the Gi protein most likely played an important role in the alcoholism pathway. To validate the impact of the Gi protein on the alcoholism pathway and provide evidence to unveil the mechanism of action of the Gi protein in ethanol-induced cells, a Western blot assay of the Gi protein was conducted among three groups (Figure 5). In contrast to the control group, the expression of the Gi protein in ethanol group was up-regulated (*p* < 0.01), whereas *Epimedium* flavonoids reversed the expression profile of the Gi protein (Figure 5). In comparison with the ethanol group, the level of Gi proteins in the flavonoids group decreased dramatically (*p* < 0.01). Additionally, the level of Gi proteins in the flavonoids group was equivalent to that in control group (*p* > 0.05). The findings of Western blot were consistent with those of proteomic analysis.

## 3. Discussion

Alcohol is attributed to 5.1% of disease burden in the world and affects various neural pathways [3,26,27]. In the clinical practice of traditional Chinese medicine, *Epimedium* was used to cure some nervous system diseases such as amnesia and neurasthenia for more than 1000 years [11,16,28]. Flavonoids are regarded as the main active ingredient in *Epimedium* [29]. It is known that some plant flavonoids (e.g., daidzin) have the potential to treat alcohol dependence [12]. In the present study, the protective effects of *Epimedium* flavonoids against ethanol-induced toxicity in neuronal cell lines were reported for the first time. Human SH-SY5Y cells could become neurons with morphological characteristics and the physiological biochemical functions of human brain neurons after differentiation induced by retinoic acid, and were used to study the pharmacological activity and mechanism of action of nervous system medicines [30,31]. *Epimedium* flavonoids were safe to RA-treated SH-SY5Y cells at a special dose (20–300 µg/mL). Hence, SH-SY5Y cells treated by RA were used as the cell model to investigate the influence of *Epimedium* flavonoids on alcoholism and to clarify their mechanism of action.

When RA-treated SH-SY5Y cells were exposed to ethanol, cell viability decreased dramatically. However, the viability remarkably increased after the intervention with *Epimedium* flavonoids. The results suggested that *Epimedium* flavonoids effectively protected SH-SY5Y cells against ethanol-induced toxicity. Additionally, this meant that *Epimedium* flavonoids might have beneficial effects on neurons with respect to alcoholism. A total of five 8-prenyl flavonol glycosides, epimedin A, epimedin B, epimedin C, epimedin F and icariin, were identified in *Epimedium* flavonoids by HPLC (Supplementary Figure S1). Among them, icariin was the most abundant flavonoid (23.20%) (Supplementary Figure S2). Icariin was reported to possess an inhibitory activity against toxicity in β-amyloid peptide-treated neurons [32]. It is implied that icariin might play a prominent role in protective effects against ethanol-induced toxicity in SH-SY5Y cells.

According to a proteomic analysis, 126 DEPs were identified between control group and ethanol group, and 122 DEPs were identified between ethanol group and flavonoids group. Among them, G proteins were found in both 126 DEPs (control group versus ethanol group) and 122 DEPs (ethanol group versus flavonoids group) and were noticeably mapped to the alcoholism pathway (Figure 4). Ethanol exposure could up-regulate the expression of the Gi protein, while *Epimedium* flavonoids reversed it to some extent (Figure 4). Usually, G proteins are considered to be an important factor in several signal transduction pathways. Many important neurotransmitters, such as acetylcholine and dopamine, usually evoke physiological responses by G proteins [33]. G proteins might be targets for some addictive drugs, and of course, they might be potential targets for some therapeutic agents [11,33,34]. Gi proteins seemed to be an important factor in the alcoholism pathway to suppress ethanol-induced toxicity. According to the pathway shown in Figure 4, we speculated that the Gi protein might protect neurons or regulate alcohol intake through inhibiting the activity of adenylyl cyclase (AC). When RA-treated SH-SY5Y cells were exposed to ethanol, the Gi protein was expressed at a high level, which might enhance the inhibition of AC by the Gi protein and reduce the phosphorylation of the cAMP response binding protein (CREB) and extracellular signal-regulated kinase 1/2 (Erk1/2), thereby blocking the reduction in alcohol intake. These findings were consistent with our previous results, where the phosphorylation of Erk1/2 in rats fed with alcohol at a concentration of 6% (*v/v*) was at a low level [35]. By contrast, when RA-treated SH-SY5Y cells were pretreated by *Epimedium* flavonoids before ethanol exposure, the expression of the Gi protein was down-regulated, which might weaken the inhibitory effect of the Gi protein on AC, and accordingly, increase the amount of cAMP, improve the phosphorylation levels of Ca^2+^/calmodulin-dependent protein kinase II (CAMK II) and CREB, and regulate the downstream BDNF-Erk1/2-PDYN axis. Thus, we speculated that *Epimedium* flavonoids might affect the expression of downstream proteins (enzymes) or their phosphorylation levels via the Gi protein. Additionally, the Gi protein might be a potential target for preventing and treating alcoholism by *Epimedium* flavonoids.

## 4. Materials and Methods

### 4.1. Chemicals and Reagents

Dulbecco’s modified Eagle’s medium (DMEM) and fetal bovine serum were bought from Hyclone Company (Logan, UT, USA). Dithiothreitol (DTT), trifluoroacetic acid (TFA), iodoacetamide (IAA) and trypsin (sequencing grade) were purchased from Promega Inc. (Madison, WI, USA). Trypsin-EDTA solution and penicillin-streptomycin liquid were bought from Beijing Solarbio S & T Co., Ltd. (Beijing, China). Anti-Gi antibody (1:1000) and goat anti-rabbit IgG (1:20000) were purchased from Bio-swamp Life Science Lab (Wuhan, China). Anti-GAPDH (1:1000) was bought from Cell Signaling Technology Company (Danvers, MA, USA). Dimethyl sulfoxide (DMSO), retinoic acid (Purity ≥ 99%), sodium dodecyl sulfate (SDS), *N*, *N*, *N*’, *N*’-tetramethyl ethylenediamine (TEMED), ammonium persulfate and 2-[4-(2-hydroxyethyl)-1-piperazinyl] ethanesulfonic acid (HEPES) were purchased from Sigma Company (St. Louis, MO, USA). MTT (3-(4,5-Dimethylthiazol-2-yl)-2,5-diphenyltetrazolium bromide) was bought from Kehao Bio-Technology Co., Ltd. (Xi’an, China). Ethanol, formic acid (FA) and acetonitrile (HPLC grade) were purchased from Fisher Chemicals (Fair Lawn, NJ, USA). Five flavonoids (epimedin A, epimedin B, epimedin C, epimedin F and icariin) (Purity ≥ 98%) and naloxone hydrochloride (Purity ≥ 99%) were bought from Shanghai Yuanye Co., Ltd. (Shanghai, China). PVDF transfer film and WBKLS0100 chemiluminescence reagent were purchased from Millipore Company (Billerica, MA, USA). BCA Protein Assay Kit (Fisher Chemicals, Fair Lawn, NJ, USA) was used for protein quantification. Tween-20, phenylmethyl sulfonyl fluoride (PMSF), acrylamide, methylene bisacrylamide (Amresco Inc., Solon, OH, USA) and protein standards (Thermo Scientific Company, Waltham, MA, USA) were applied to protein electrophoresis.

### 4.2. Preparation of Epimedium Flavonoids

*Epimedium brevicornu* was collected from Shaanxi Province of China. Flavonoids were isolated from *Epimedium* by ultrasonic extraction as described previously [29]. Briefly, *Epimedium* leaves were dried naturally, and then ground into powders. The leaf powders were ultrasonically extracted using a JPCQ0328 ultrasonic instrument (Wuhan Jiapeng Electronics Co., Ltd., Wuhan, China) under the following conditions: ethanol concentration of 63% (*v*/*v*), ratio of liquor to solid of 70:1 (mL/g), maceration time of 30 min, extraction duration of 39 min, and extraction temperature of 38 °C. After ultrasonication, the mixture was centrifuged at 4000 rpm for 10 min, and the supernatants were filtered through 0.45 μm microporous membranes. The filtrates were concentrated using RE-52 rotary evaporator (Anting Scientific Instrument Co., Ltd., Shanghai, China), and then lyophilized using FDU-1200 vacuum freeze drier (Tokyo Rikakikai Co., Ltd., Tokyo, Japan).

### 4.3. Qualitative and Quantitative Analyses of Epimedium Flavonoids

*Epimedium* flavonoids were qualitatively and quantitatively analyzed according to Pharmacopoeia of the People’s Republic of China [29,36]. Flavonoids were identified by color reaction with magnesium powder and hydrochloric acid. Additionally, contents of epimedin A, epimedin B, epimedin C, epimedin F and icariin were determined using Breeze 1525 HPLC system equipped with Breeze 2487 ultraviolet-visible detector (Waters Corporation, Milford, MA, USA) and ZORBAX SB-C8 reverse-phase analytical column (Agilent Technologies, Santa Clara, CA, USA) [11,28]. The results of color reaction with magnesium powder and hydrochloric acid confirmed the chemical nature of *Epimedium* flavonoids. Additionally, HPLC analysis showed that the major constituents of *Epimedium* flavonoids were icariin, epimedin A, epimedin B, epimedin C and epimedin F (Supplementary Figure S1).

### 4.4. Cell Culture and Experimental Treatment

Cell experiment was conducted according to Wernicke et al. (2010) [30]. The experiment was approved by the Advisory Ethics Committee of International Joint Research Center of Shaanxi Province for Food and Health Sciences. Briefly, SH-SY5Y cells were cultured in Dulbecco’s modified Eagle’s medium supplemented with fetal bovine serum. The undifferentiated cells were trypsinized and induced with RA (final concentration of 10 µM) for 72 h. The differentiated SH-SY5Y cells with characteristics of neurons were subjected to ethanol exposure and drug treatment. The experiment contained 4 groups: control group, ethanol group, naloxone hydrochloride group and flavonoids group. In ethanol group, cells were treated using ethanol. In naloxone hydrochloride group, cells were treated using naloxone hydrochloride followed by ethanol. In flavonoids group, cells were treated using various concentrations of *Epimedium* flavonoids followed by ethanol. In control group, cells were not treated with ethanol, naloxone hydrochloride or *Epimedium* flavonoids. In all groups, RA was always present in the medium.

### 4.5. Cell Viability Assay

SH-SY5Y cell viability was determined by MTT assay according to the manufacturer’s protocol [5,9]. Briefly, MTT was dissolved in phosphate-buffered saline (PBS). MTT solutions were added to each well containing SH-SY5Y cells and incubated for 4 h. The live cells reduced MTT (yellow) to insoluble formazan crystals (purple). Then, DMSO was added to solubilize the crystals. Additionally, the absorbance was measured at 490 nm using Enspire multifunctional plate reader (PerKinElmer Company, Waltham, MA, USA). Cell viability was calculated by the following formula:

I (%) = (A_s_ − A_b_)/(A_c_ −A_b_) × 100%

where I (%) represented cell viability, A_s_ represented the absorbance of ethanol group, naloxone hydrochloride group, or flavonoids group, A_c_ represented the absorbance of control group, and A_b_ represented the absorbance of the blank (absence of cells). Data were expressed as means ± SD.

### 4.6. Isolation and Quantification of Proteins

Proteins were extracted by the method reported by Lu et al. (2016) [25] with some modifications. Cells were collected by a scraper with PBS, and then centrifuged at 1000 rpm for 5 min. Lysis solution (7 M urea, 4% SDS, 30 mM HEPES, 1 mM PMSF, 2 mM EDTA, 10 mM DTT, and 1× protease inhibitor) was added to cell samples, and proteins were ultrasonically extracted for 10 min on ice. The resulting mixture was spun, and the supernatant was collected as total proteins. Total proteins were subjected to proteomic analysis and Western blot analysis. Total proteins were treated by DTT, IAA and cold acetone in turn. Firstly, DTT was added to total proteins and the mixture was incubated at 55 °C. Secondly, IAA was added immediately, and the mixture was placed in the dark for 1 h. Thirdly, four volumes of cold acetone were added, and the mixture was placed at −20 °C for 3 h. Afterwards, the mixture was centrifuged, and the precipitate was collected and washed by acetone and then ethanol. The precipitate was suspended in ammonium bicarbonate solution for further proteomic analysis.

### 4.7. Proteomic Procedure

Proteomic procedure included enzymatic hydrolysis, purification and mass spectrometric detection [25]. Regarding enzymatic hydrolysis, proteins solution was digested with trypsin at 37 °C. After 16 h, TFA solution was added to stop enzymatic hydrolysis. Regarding purification, TFA solution and acetonitrile were, respectively, used to activate and balance Sep-Pak C18 desalting column (Waters Corporation, Waltham, MA, USA). After salt and other small molecules were removed, peptides were collected and freeze-dried. Regarding mass spectrometric detection, peptides were dissolved and then separated by NanoAcquity ultra-high pressure nanoscale liquid chromatographic system (Waters Corporation, Waltham, MA, USA) equipped with Acclaim PepMap C18 columns (Thermo Scientific Company, Waltham, MA, USA). Higher energy C-trap dissociation (HCD)-mass spectrometry (MS) was applied for the detection of peptides using Q Exative mass spectrometer (Thermo Scientific Company, Waltham, MA, USA).

### 4.8. Bioinformatic Analysis

MaxQuant computational platform (Version 1.5.8.3) was used to analyze the results of mass spectrometry using Uniprot–SwissProt resource as the database and *Homo sapiens* as the reference species [37]. Proteins were quantified by intensity-based absolute quantification (iBAQ) [38]. The online database DAVID 6.8 and gene ontology tool were applied to functional classification and annotation analysis [39,40]. Pathway analysis of DEPs was performed using Kyoto Encyclopedia of Genes and Genomes website [41].

### 4.9. Western Blot Analysis

Western blot analysis was performed according to Wang et al., (2012) [35]. Total proteins were separated by SDS-polyacrylamide gel electrophoresis, and then transferred to polyvinylidene fluoride membrane. The membrane was treated with nonfat dry milk and Tween-20 in PBS, and incubated with rabbit anti-Gi antibody (1:1000) or rabbit anti-GAPDH (1:1000) for 12 h at 4 °C. Then, the membrane was washed 2–4 times with PBS containing 0.05% Tween-20 and incubated with secondary antibody (i.e., goat anti-rabbit IgG) (1:20,000) labeled by horseradish peroxidase (HRP) for 1 h. The bands were visualized by enhanced chemiluminescence assay (ECL) using Fusion Fx spectra imaging system (Vilber Lourmart, Marne-la-Valleé, France). The relative expression levels of proteins were analyzed using GAPDH as internal reference.

### 4.10. Statistical Analysis

All experiments were repeated 2–5 times, and all data were expressed as means or means ± SD. The statistical significance was evaluated using Microsoft Excel and SPSS software (SPSS Inc. Chicago, IL, USA). One-way ANOVA analysis was used for comparison between groups. *p* < 0.05 and *p* < 0.01 represented statistically significant and very significant, respectively.

## 5. Conclusions

*Epimedium* flavonoids notably exerted a protective potential against ethanol-induced toxicity in retinoic acid-treated SH-SY5Y cells. The mechanism underlying their protective effects was explored by a label-free proteomic approach combined with bioinformatic analysis for the first time. The impact of the Gi protein on the alcoholism pathway was validated by a Western blot assay. Hundreds of DEPs were identified and classified as biological process, cellular component and molecular function. The expression of Gi protein was up-regulated under the influence of ethanol, whereas its expression was down-regulated after the intervention with *Epimedium* flavonoids. The Gi protein seemed to be an important factor in the alcoholism pathway for suppressing ethanol-induced toxicity of SH-SY5Y cells.

## Data Availability

The data presented in this study are available on request from the corresponding author.

## Supplementary Materials

Figure S1: HPLC profile of *Epimedium* flavonoids; Figure S2: Chemical structures of epimedin A, epimedin B, epimedin C, epimedin F and icariin.
