# Supplementary material for: Proteomic Analysis of Protective Effects of Epimedium Flavonoids against Ethanol-Induced Toxicity in Retinoic Acid-Treated SH-SY5Y Cells
Source: Molecules. 2022 Feb 2;27(3):1026. doi: 10.3390/molecules27031026 (PMC8838442; doi:10.3390/molecules27031026)
Supplement: Supplementary file 1 [file molecules-27-01026-s001.zip › molecules-1557491-supplementary.pdf]

SUPPLEMENTARY MATERIALS

FIGURE LEGENDS

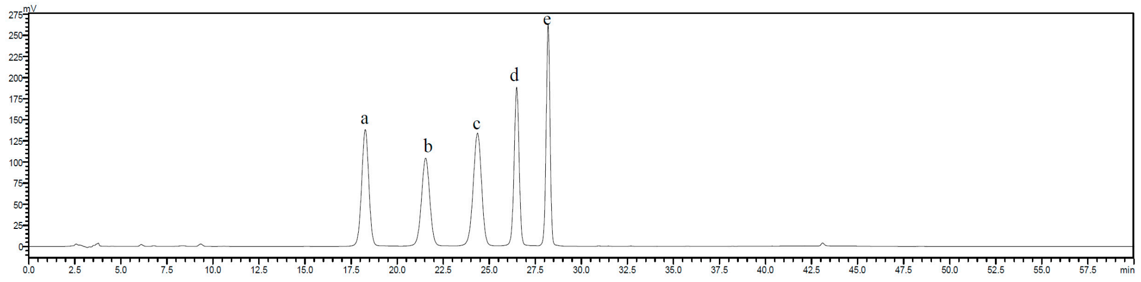

(A)

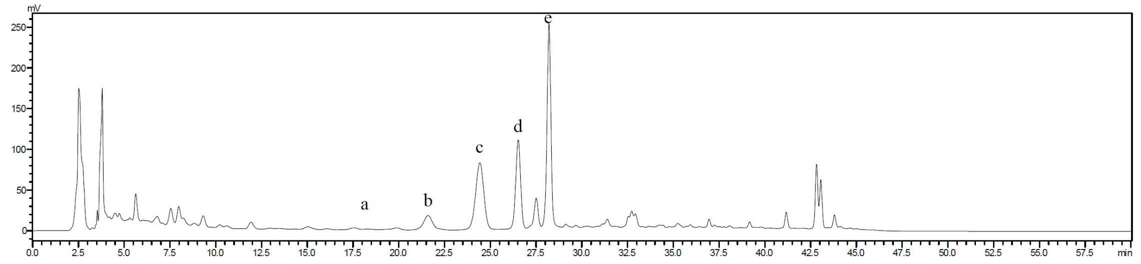

(B)

Figure S1. HPLC profile of *Epimedium* flavonoids. (A) Standards; (B) *Epimedium* flavonoids sample. The peaks a, b, c, d and e represent epimedin F, epimedin A, epimedin B, epimedin C and icariin, respectively. Peaks without a marker were not identified. The *x* and *y* coordinates represent the retention time (min) and response value of HPLC-detector (AU), respectively.

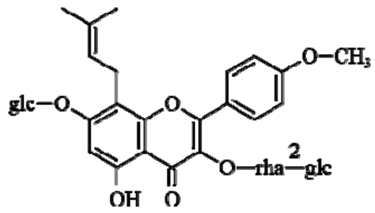

Epimedin A

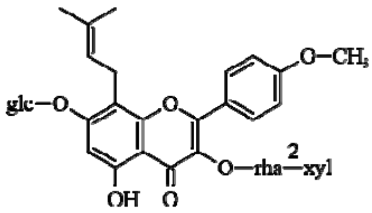

Epimedin B

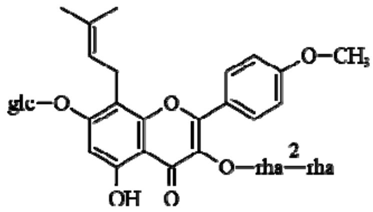

Epimedin C

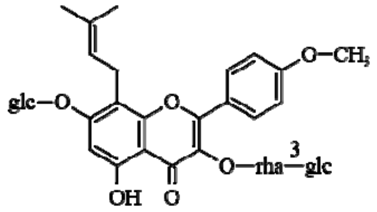

Epimedin F

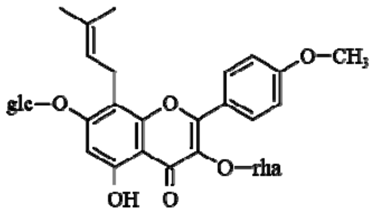

Icariin

Figure S2. Chemical structures of epimedin A, epimedin B, epimedin C, epimedin F and icariin.
